# Causes of maladaptation

**DOI:** 10.1111/eva.12844

**Published:** 2019-08-13

**Authors:** Steven P. Brady, Daniel I. Bolnick, Amy L. Angert, Andrew Gonzalez, Rowan D.H. Barrett, Erika Crispo, Alison M. Derry, Christopher G. Eckert, Dylan J. Fraser, Gregor F. Fussmann, Frederic Guichard, Thomas Lamy, Andrew G. McAdam, Amy E.M. Newman, Antoine Paccard, Gregor Rolshausen, Andrew M. Simons, Andrew P. Hendry

**Affiliations:** ^1^ Biology Department Southern Connecticut State University New Haven CT USA; ^2^ Department of Ecology and Evolutionary Biology University of Connecticut Mansfield CT USA; ^3^ Departments of Botany and Zoology University of British Columbia Vancouver BC Canada; ^4^ Department of Biology McGill University Montréal QC Canada; ^5^ Quebec Centre for Biodiversity Science, Stewart Biology McGill University Montréal QC Canada; ^6^ Redpath Museum McGill University Montréal QC Canada; ^7^ Department of Biology Pace University New York NY USA; ^8^ Département des sciences biologiques Université du Québec à Montréal Montréal QC Canada; ^9^ Department of Biology Queen's University Kingston ON Canada; ^10^ Department of Biology Concordia University Montréal QC Canada; ^11^ Département de sciences biologiques Université de Montréal Montréal QC Canada; ^12^ Marine Science Institute University of California Santa Barbara CA USA; ^13^ Department of Integrative Biology University of Guelph Guelph ON Canada; ^14^ McGill University Genome Center Montréal QC Canada; ^15^ Senckenberg Biodiversity and Climate Research Centre (SBiK‐F) Frankfurt am Main Germany; ^16^ Department of Biology Carleton University Ottawa ON Canada

**Keywords:** adaptation, fitness, global change, maladaptation

## Abstract

Evolutionary biologists tend to approach the study of the natural world within a framework of adaptation, inspired perhaps by the power of natural selection to produce fitness advantages that drive population persistence and biological diversity. In contrast, evolution has rarely been studied through the lens of adaptation's complement, maladaptation. This contrast is surprising because maladaptation is a prevalent feature of evolution: population trait values are rarely distributed optimally; local populations often have lower fitness than imported ones; populations decline; and local and global extinctions are common. Yet we lack a general framework for understanding maladaptation; for instance in terms of distribution, severity, and dynamics. Similar uncertainties apply to the causes of maladaptation. We suggest that incorporating maladaptation‐based perspectives into evolutionary biology would facilitate better understanding of the natural world. Approaches within a maladaptation framework might be especially profitable in applied evolution contexts – where reductions in fitness are common. Toward advancing a more balanced study of evolution, here we present a conceptual framework describing causes of maladaptation. As the introductory article for a Special Feature on maladaptation, we also summarize the studies in this Issue, highlighting the causes of maladaptation in each study. We hope that our framework and the papers in this Special Issue will help catalyze the study of maladaptation in applied evolution, supporting greater understanding of evolutionary dynamics in our rapidly changing world.

It may metaphorically be said that natural selection is daily and hourly scrutinising, throughout the world, the slightest variations; rejecting those that are bad, preserving and adding up all that are good; silently and insensibly working, whenever and wherever opportunity offers, at the improvement of each organic being in relation to its organic and inorganic conditions of life. Darwin (1872, p. 65)

## 1 PREAMBLE AND INTRODUCTION TO A SPECIAL ISSUE ON MALADAPTATION

**Figure 1:**
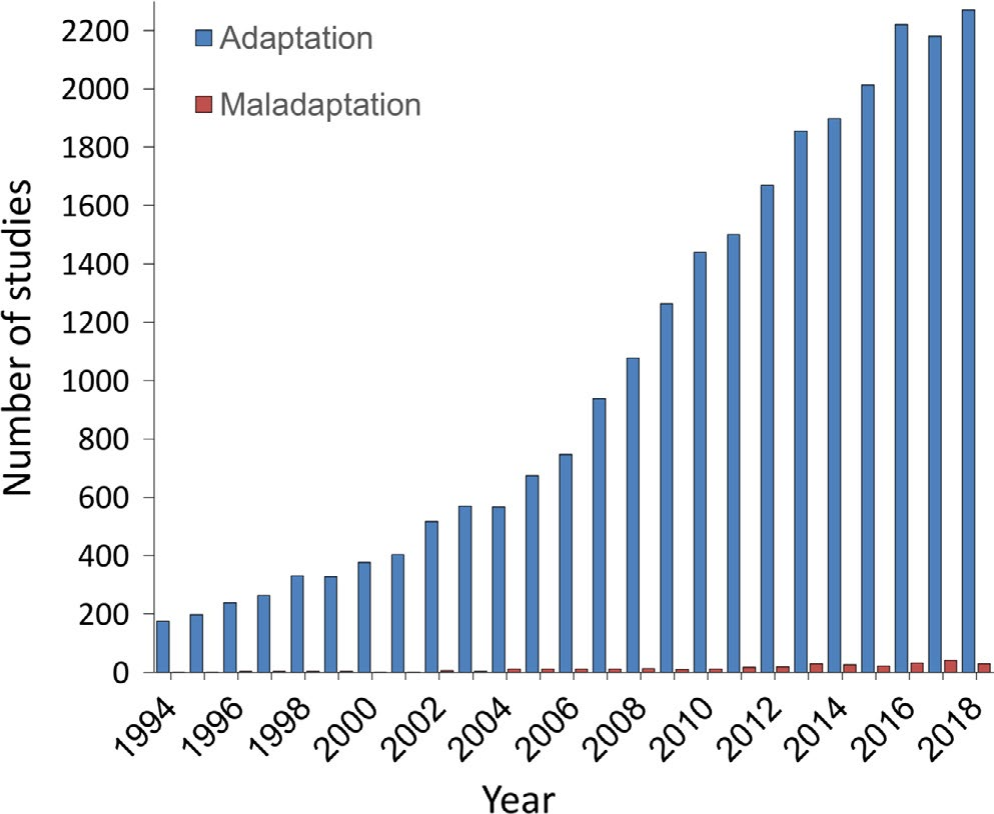
Number of evolutionary studies referring to adaptation versus maladaptation. Data were obtained by searching Web of Science Core Collections on July 16, 2019. Studies reporting adaptation (blue bars) were identified by searching on *“*evolution* and (ecolog* or biol*) and (adapt*)” whereas studies reporting maladaptation (red bars) were identified by searching on “evolution* (and ecolog* or biol*) and maladapt*”

Generations of evolutionary biologists have emphasized adaptation, inspired by the power of natural selection to scrutinize and improve organisms' fit to their environment. Yet, biologists have long been aware of the other side of this coin: For selection to act on a population, individuals must be some distance from the adaptive optimum. Maladaptation is thus just as deserving a focus as adaptation (Brady et al., 2019a). Although maladaptation is neither a new concept nor an unexpected outcome, it is often underemphasized relative to its complement, adaptation. Much like “Rubinʼs vase” — the illusion in which we tend to see a single image where two are present (i.e., a vase and two faces) — we biologists tend to see evolution in terms of its successes (adaptation) rather than its failings (maladaptation; Figure 1), despite both outcomes being present (Crespi, 2000; Hendry & Gonzalez, 2008). As with seeing the complete picture in Rubin's vase, understanding biology involves looking at evolution from both perspectives. To encourage a greater focus on maladaptation, this Special Issue presents a collection of research studies investigating evolution and its consequences through the lens of maladaptation.

Why should this imbalance matter? By one view, maladaptation might simply be considered the flip side of adaptation. In this case, a focused approach to maladaptation might offer little or no advantage over approaches focused on adaptation. Alternatively, maladaptation might represent something more complex, nuanced, or insightful about evolution and ecology. If maladaptation is to be worth studying in its own right, it should have causes or consequences that are best articulated or studied in terms of maladaptation rather than just the lack of adaptation. For instance, we are keenly aware of local adaptation among populations, and natural selection's role as the driving force. But rarely is a lack of local adaptation discussed in terms of maladaptation (e.g., the distance from adaptive optima), and the various forces at play. This Special Issue makes clear the many practical concerns (e.g., conservation, agriculture, domestication, evolutionary medicine) that are most clearly seen in light of maladaptation (e.g., Derry et al., 2019; Martinossi‐Allibert, Thilliez, Arnqvist, & Berger, 2019; Walters & Berger, 2019).

To help clarify our conception of maladaptation, we begin this Special Issue with a consideration of the meaning and multifarious causes of maladaptation (i.e., what prevents populations from being well adapted). The conceptual basis for maladaptation is elusive and confusing to many, resulting in a muddled treatment and discourse. What outcomes constitute maladaptation and how do we diagnose it? For instance, must maladaptation entail an evolutionary (genetic) change, or is it equally a function of environmental change? Is a population maladapted if habitat degradation caused population decline? Is a trait considered maladaptive if it contributes to positive population growth but is suboptimal relative to other phenotypes?

We, of course, are not the first to ponder these questions (Crespi, 2000; Dobzhansky, 1968a, 1968b; Endler, 1986; Hendry & Gonzalez, 2008), and competing opinions still circulate. Rather than argue for a single universal definition, Brady et al. (2019a) discussed the various meanings of maladaptation, the different metrics (absolute or relative fitness) and reference points, and how different definitions are best suited to different research contexts. They further advocated for joint consideration of absolute and relative fitness in studies of (mal)adaptation, suggesting that “absolute maladaptation” occurs when fitness is less than replacement (W<1) whereas “relative maladaptation” occurs when fitness is less than some other point of comparison (e.g., $W<W_{\max}$). Here, we do not further revisit these various definitions. Rather, our aim in this paper is to provide a framework for the causes of maladaptation, illustrated using an archery metaphor of arrows and targets. We hope that this framework clarifies the many facets of maladaptation and that the articles in this Special Issue will inspire new work helping to balance our understanding of maladaptation with that of adaptation.

## 2 DESPITE OUR PREOCCUPATION WITH ADAPTATION, MALADAPTATION ACTUALLY IS ALL AROUND US

Scientists and naturalists have long marveled at adaptation, placing considerable focus on the power of natural selection to act on trait variation that results in adaptation and diversification (Cain, 1989; Darwin, 1859; Endler, 1986; Kawecki & Ebert, 2004; Nosil, Crespi, & Sandoval, 2002). After all, adaptation is all around us. Populations evolve adaptively as environments change (Burger & Lynch, 1995); residents often have higher fitness than immigrants (Hereford, 2009; Leimu & Fischer, 2008); traits differ across species' ranges in predictable ways (Chuang & Peterson, 2016); and invasive species can rapidly colonize and adapt to new environments (Colautti & Barrett, 2013; Phillips, Brown, Webb, & Shine, 2006).

Our fixation with the adaptive side of biology is evident in the literature: Each year, hundreds of published studies refer to adaptation, whereas fewer than 40 refer to maladaptation (Figure 1). It seems adaptation is what we have come to expect in nature, and when we look for adaptation, we often find it. But what would we learn if instead we looked for maladaptation (Brady, 2013, 2017; Crespi, 2000; Gould & Lewontin, 1979; Hendry & Gonzalez, 2008; Hereford & Winn, 2008; Rogalski, 2017)? For instance, quantitative syntheses and meta‐analyses of reciprocal transplant experiments have found that the classic signature of local adaptation is present in about 70% of the contrasts, meaning that it was absent (or undetected) 30% of the time (Hereford, 2009; Leimu & Fischer, 2008). That such studies are applied nonrandomly to contexts where adaption is expected a priori, coupled with publication bias (i.e., “file drawer problem”), suggests that maladaptation might be even more prevalent than this 70:30 ratio might imply. Another piece of evidence for the ubiquity of maladaptation is that every instance of detectable natural selection could be viewed in terms of selection against maladapted individuals (Barton & Partridge, 2000; Haldane, 1957), given that well‐adapted populations should have phenotypes near the optimum and thus not experience much selection (Haller & Hendry, 2014). Yet, reviews suggest that this is rarely the case. For instance, Estes and Arnold (2007) calculated that population mean phenotypic values were at least one standard deviation from the inferred optimum in 64% of cases. Maladaptation is also reflected by frequent periods of population decline and local extinctions (Hanski, 1990; Harrison, 1991); the occurrence of sink populations (Furrer & Pasinelli, 2016); and the reduction of population fitness near species' range limits (Angert & Schemske, 2005; Hargreaves, Samis, & Eckert, 2013; Lee‐Yaw et al., 2016). Finally, and perhaps most telling, most of the populations and species that at one time existed on earth have gone extinct (Kunin & Gaston, 1997; Novacek & Wheeler, 1992). In short, despite our penchant for studying adaptation, maladaptation is an important and common result of evolution.

Not only has maladaptation always been a fundamental aspect of life's history, it might be ever more prevalent and important as human activities dramatically and rapidly change environmental conditions. That is, as a result of human‐induced environmental change, many populations are declining, extirpations and extinctions are becoming increasingly common (Ceballos, Ehrlich, & Dirzo, 2017; Dirzo et al., 2014), and evidence for local maladaptation is mounting (Brady, 2013; Rogalski, 2017; Rolshausen et al., 2015). Thus, understanding how maladaptation will impact populations and their ecosystems will require careful consideration of the processes that generate maladaptation and the dynamics that ensue.

## 3 MALADAPTATION: WHEN FITNESS MISSES THE MARK

One reason maladaptation can be so common is that it can have many causes. The “Anna Karenina principle” expressed the notion that there are many paths to failure, many ways a system can be broken. To illustrate these many paths to maladaptation, we use the framework of a fitness surface, the relationship between individuals' trait value and absolute fitness (e.g., lifetime reproductive success).

The mean fitness of a population ($\overset{¯}{W}$) depends on both its trait distribution and the individual fitness landscape: that is, the relation between individual trait values and individual fitnesses (Lande, 1976; Simpson, 1944). For illustration, consider a fitness landscape with symmetric stabilizing selection, using a single trait for simplicity: The fitness of individuals with trait value *x* can be expressed as.

(1) $$W_{x}=W_{\max}*\exp(-\left(x_{i}{-\theta )}^{2}/\omega \right)$$

where θ is the optimal trait value and ω is the strength of stabilizing selection (here, we assume there is a single optimum). Integrating across the trait distribution, population mean fitness is then $\int_{-\infty }^{\infty }W_{x}p\left(x\right)dx$, where $p\left(x\right)$ is the frequency of trait x in the population. This formulation assumes there is a single optimum so that selection would eventually eliminate all variation (e.g., the optimal trait variance is ν=0). This assumption can be relaxed: For example, frequency‐dependent interactions (not captured in Equation 1) can lead to cases where population mean fitness is maximized for population trait variances greater than zero (e.g., there is an optimal trait variance ν>0). Thus, for a population with a quantitative trait *x* ~ *N*($\overset{¯}{x},\sigma_{x}^{2}$), three distinct problems can cause maladaptation: (a) The trait mean ($\overset{¯}{x})$ can be displaced from the optimal trait value (θ); thus, $\overset{¯}{x}\neq \theta$; (b) the trait variance ($\sigma_{x}^{2})$ can deviate from the optimal variance; thus, $\sigma_{x}^{2}\neq \nu$; or (c) the maximum achievable fitness can be low (e.g., $W_{\max}$ < 1), even when $\overset{¯}{x}=\theta$ and $\sigma_{x}^{2}=\nu$. Note that in the formula for mean fitness, above, optimal variance is implicitly assumed to be zero (e.g., stabilizing selection should eventually erode all variation).

Each of these three problems can arise from one or more of three events: (a) a change in the trait distribution (observed mean or variance) due to genetic load (e.g., genetically based difference between the mean phenotype and the optimum phenotype) or maladaptive plasticity ($\frac{d\overset{¯}{x}}{\mathrm{dt}}$ or $\frac{d\sigma_{x}^{2}}{\mathrm{dt}})$, (b) a change in the fitness landscape (optimal mean or variance) due to an exogenous environmental change $(\frac{d\theta }{\mathrm{dt}}$, $\frac{d\nu }{\mathrm{dt}}$, or $\frac{dW_{\max}}{\mathrm{dt}}$), or (c) an eco‐evolutionary or eco‐plasticity feedback in which the focal populations' abundance, evolution, or plasticity alters its fitness landscape; numerous possible feedback loops exist, for instance, when population dynamics alter the optimal trait value, in turn causing population mean trait value to evolve, in turn influencing population dynamics, and so on $\left(\frac{d\theta }{\mathrm{dN}}\to \frac{d\overset{¯}{x}}{d\theta }\to \frac{d\text{N}}{d\overset{¯}{x}}\to \ldots \right).$ Taking in combination these three causal events with the three sources of reduced fitness from above (i.e., $\overset{¯}{x}\neq \theta ;\;\sigma_{x}^{2}\neq \nu ;\;W_{\max}$ < 1), we identify nine distinct causes of maladaptation, which are nevertheless not mutually exclusive.

## 4 NINE CAUSES OF MALADAPTATION USING AN ARCHERY METAPHOR

**Figure 2:**
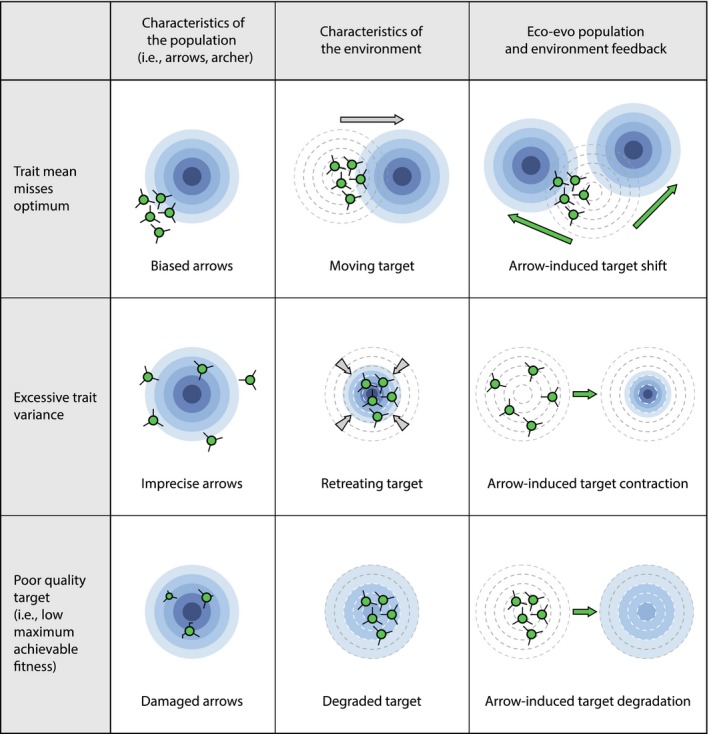
Scenarios of maladaptation. Nine scenarios are illustrated using an archery metaphor of arrows and targets. In each scenario, arrows indicate representative individuals of the population while the target represents the fitness landscape. Rows indicate trait–fitness landscape scenarios that can generate maladaptation. Columns indicate various causes of the scenarios, involving either change in the focal population (left), change in the environment (middle), or eco‐evolutionary/eco‐plasticity feedbacks in which the focal population's evolution or dynamics alter the fitness landscape

**Figure 3:**
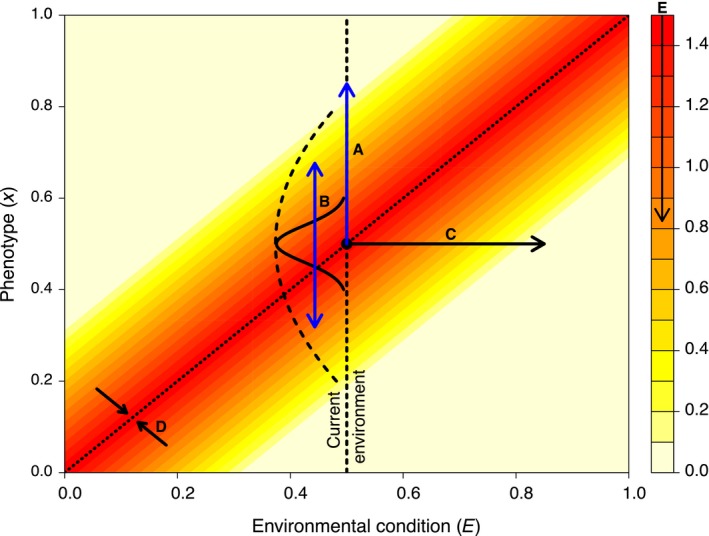
A conceptual fitness surface showing various ways for mean absolute fitness to decline. Fitness is indicated by heat map colors and is shown in relation to environmental condition (*x*‐axis) and phenotype value (*y*‐axis). Under conditions shown, there exists a range of phenotype and environment values that confer maximal fitness. Scenarios causing maladaptation are represented in terms of trait distribution change (blue arrows) and environmental change (black arrows). For trait distribution change, maladaptation can arise through (A, *biased arrows*) resulting from change in trait mean ($\frac{\text{d}\overset{¯}{x}}{\text{d}t}$) that reduces mean fitness or (B, *imprecise arrows*) increasing trait variation ($\frac{\text{d}\;\;\;\text{var}(x)}{\text{d}t}$) e.g., due to immigration, assortative mating, mutation, maladaptive plasticity), which increases variance in fitness and thereby reduces mean fitness). For environmental change, maladaptation can arise when (c, *moving target*) the environmental value changes ($\frac{\text{d}E}{\text{d}t}$), (D, *retreating target*) the fitness peak narrows ( $\frac{\text{d}\;\;\;\text{var}(E)}{\text{d}t}$; e.g., due to increased competition or niche contraction) resulting in stronger stabilizing selection which in turn increases variance in fitness and thereby reduces mean fitness, or (E, *degraded target*) the environmental quality decreases ($\frac{\text{d}W_{\max}}{\text{d}t}$).

For an intuitive metaphor to convey the nine distinct scenarios of maladaptation, we depict the fitness landscape as an archery target, with rings representing topographic contour lines of the landscape. This metaphor necessarily implies two traits (*x*,*y*), but is analogous to the one‐trait landscape described above. We use arrows to represent individuals with particular phenotypes (for clarity, only a representative subset of arrows is shown in each case; Figure 2). With this metaphor, we start by describing maladaptation arising from changes in population trait distributions (column 1 in Figure 2) and then move to maladaptation arising from changes in the environment (column 2), and then to maladaptation arising from eco‐evolutionary or eco‐plasticity feedbacks (column 3). We further illustrate each of these nine causes with reference to a fitness landscape depicted as a heat map of individual fitness for each possible trait–environment combination (Figure 3).

### 4.1 Biased (but potentially precise) arrows

A variety of evolutionary processes can drag a population's mean trait value away from the local optimum, thus missing the adaptive target (one‐tailed blue arrow “A” in Figure 3). These processes include mutation (although typically having only a very small effect on trait means at any point in time, and more likely to increase than bias variance) (e.g., Kibota & Lynch, 1996); genetic drift (although perhaps severe only in small populations) (e.g., Newman & Pilson, 1997); gene flow from other populations (or other times, such as in the case of seed banks from prior generations) (Falahati‐Anbaran, Lundemo, & Stenøien, 2014; Garant, Forde, & Hendry, 2007; Paul, Sheth, & Angert, 2011); trade‐offs, whether functional (e.g., due to antagonistic pleiotropy) or not (e.g., genetic linkage); and plasticity (e.g., imperfect cue sensing). As one example, populations of the walking stick insect *Timema cristinae* with higher rates of immigration experience higher degrees of maladaptation measured in terms of the frequency of a less‐cryptic morph (Bolnick & Nosil, 2007). Shifts in mean trait value also can be caused by maladaptive plasticity generated by environmental stressors or other novel environmental changes. Also, some traits can be maladaptive as a result of selection on correlated traits (Hutchings, 2005), and some traits can be maladaptive at certain times, such as when optima shift through the life cycle (Schluter, Price, & Rowe, 1991), or in certain contexts, such as predator avoidance versus competition (Nuismer & Doebeli, 2004). Of course, gene flow, mutation, and plasticity are not all bad—because these processes also contribute genetic and phenotypic variation that could help a currently maladapted population climb toward a new adaptive peak (Garant et al., 2007). Continuing the metaphor, biased arrows can become accurate if the target shifts in the direction of the bias.

### 4.2 Imprecise (but potentially unbiased) arrows

When populations are subject to stabilizing selection, high phenotypic variance generates a “load” that reduces mean fitness (Burt, 1995; Hansen, Carter, & Pélabon, 2006). That is, even if a population's mean trait value is exactly optimal, most individuals will be some distance from that optimum. As a result, $\overset{¯}{W}<W_{\max}$. This potential excess trait variance (two‐tailed blue arrow “B” in Figure 3) can be due to maladaptive plasticity (e.g., developmental noise due to environmental stress) or genetic noise due to mutation, gene flow, recombination/segregation, and assortative mating. Over time, persistent stabilizing selection should reduce this maladaptive genetic variation, and its sources (e.g., reducing migration rates), but this process can be slow and ultimately unable to eliminate the processes generating genetic load. Also, following from the above, increases in variance can be beneficial in the longer term by providing the raw material for future adaptation: That is, imprecise arrows might be more likely to at least sometimes hit moving (or other existing) targets.

On the flip side, maladaptation could be caused by *too little* trait variance, which would instead be akin to overly precise arrows in the metaphor (not illustrated in Figure 2). In some contexts, for instance, increased trait variation can be beneficial, such as through frequency dependence or bet hedging across temporal variation (Meyers & Bull, 2002; Roulin, 2004; Simons, 2009). In such cases, mean fitness—or at least overall population size—would be higher if more individuals had nontypical phenotypes. Also, fine‐scale environmental heterogeneity is ubiquitous, such as light flecks on a forest floor (Endler, 1993), north/south‐facing exposures (Bennie, Hill, Baxter, & Huntley, 2006), or changing light and substrate with depth in a lake or ocean (Partensky, Blanchot, Lantoine, Neveux, & Marie, 1996; Stocker, 2012). Populations that span such heterogeneity (akin to multiple targets in range) might evolve plasticity, generalist phenotypes, or a diversity of individual specialists (Bolnick et al., 2002; Rueffler, Van Dooren, Leimar, & Abrams, 2006). These cases where increased trait variance would be beneficial often invoke high competition among individuals with common phenotypes (Bolnick, 2001).

### 4.3 Damaged arrows

The above effects imply some noteworthy mismatch between actual and optimal trait means and variances. However, reduced fitness can also be caused by overall organismal degradation independent of any particular phenotype. For example, weakly deleterious alleles can accumulate in small populations where drift is strong and selection is relatively inefficient (e.g., mutation–selection–drift balance; one‐tailed blue arrow “A” in Figure 3). A well‐known example in asexual populations is “Mueller's ratchet”; yet, such mutational meltdown also can occur in sexual organisms. This process might be most likely in environments that increase rates of DNA damage or mutation, with clear examples being high‐altitude sites with excessive UV radiation, urban sites with pollution, and nuclear accident sites with ionizing radiation (Häder & Sinha, 2005; Møller & Mousseau, 2015; Yauk, Fox, McCarry, & Quinn, 2000). In such environments, residents with genomic damage can have lower fitness than individuals from other (less damaging) environments when evaluated in the resident environment. For *damaged arrows*, optimal traits (e.g., the ability to repair damaged DNA) might be unattainable, constituting a target shift (see *moving target* below) beyond the range of arrows.

The effects just described can occur without ongoing adaptation; yet, almost cruelly, they can also be a side effect of very strong ongoing adaptation if strong “hard” selection (Reznick, 2015) reduces population sizes so much that drift or inbreeding depression becomes problematic (Falk, Parent, Agashe, & Bolnick, 2012; Wade, 1985). In such cases, the fitness gains owing to adaptation can be more than offset by coincidental fitness declines owing to the resulting population declines and bottlenecks (which can also impair future adaptation by reducing variation). Such effects do require exceptionally strong selection to induce strong drift (Gillespie, 2010), and so are unlikely to be general.

### 4.4 Moving target

The preceding three scenarios focused on mechanisms of organismal change resulting in suboptimal phenotypes or other forms of genetic load, all potentially independent of environmental change. The next three scenarios (starting here with “moving target”) instead focus on environmental changes generating maladaptation, potentially independent of phenotypic or genetic change. Most obviously, the optimal phenotype can change with environmental conditions (black arrow “C” pointing to the right in Figure 3), which can increase the distance of the optimum phenotype from current phenotypes, which should decrease mean fitness. If adaptive genetic variation is sufficient, the population might rapidly adapt to the new optimum, in which case maladaptation will be transient (see Brady et al., 2019a). However, substantial lags in adaptation can arise if (a) the population lacks sufficient genetic variation in the appropriate trait dimensions (Hine, McGuigan, & Blows, 2014; Kirkpatrick, 2009); (b) the environmental change is too large and/or abrupt (Bell & Collins, 2008; Burger & Lynch, 1995; Chevin, Lande, & Mace, 2010); (c) the environment change is ongoing—whether directional or fluctuating (Lively, Craddock, & Vrijenhoek, 1990); or (d) the demographic costs of adaptation dramatically reduce population size (Uecker, Otto, & Hermisson, 2014).

As a special case of the moving target, the environment might change in a duplicitous way, triggering a behavioral response that would have been adaptive in a past context but is maladaptive in the new (often human‐modified) context. Such “evolutionary traps” have been reported across a wide variety of contexts, from reflected light inducing insects to oviposit on inappropriate structures, such as glass windows, to seabirds ingesting floating plastics and other pieces of garbage that resemble typical food sources (Robertson, Rehage, & Sih, 2013).

### 4.5 Retreating target

Environmental change can alter the intensity of stabilizing selection (i.e., width of the adaptive peak) and, thereby, mean fitness in a population whose mean phenotype remains well adapted. For instance, a narrowing of the adaptive peak increases stabilizing selection (Figure 3 arrows “D” pointing toward the line of unity representing optimal phenotypes) and thereby increases genetic load for a given phenotypic distribution. The resulting mismatch between observed and optimal trait variances is thus an environment‐driven parallel to the above phenotype‐driven “imprecise arrow” scenario. As one example, a loss of environmental complexity, or an increase in “ecological simplification” (Peipoch, Brauns, Hauer, Weitere, & Valett, 2015), might reduce suitable niche space (Kohn & Leviten, 1976) and thereby transform a plateau of optimality into a steepened peak of optimality.

On the other hand, environmental change could broaden the range of trait values conferring high fitness or even generate additional trait optima if, for instance, new food resources appear that provide alternative foraging opportunities for individuals with suitable trophic morphology (Martin & Wainwright, 2013). When several discrete optima exist simultaneously (not illustrated in Figure 3), populations might evolve a discrete polymorphism in which trait modes correspond to the alternative optima. Often, quantitative genetic inheritance in sexual organisms smooths the population's trait distribution, ensuring that maladaptive intermediate variants persist and stably occupy the fitness valleys between optima (Rosenzweig, 1978). In some cases, however, assortative mating can help to maintain coadapted traits associated with polymorphisms (e.g., Lancaster, McAdam, Hipsley, & Sinervo, 2014).

### 4.6 Degraded target

Environmental change can reduce population mean fitness even if the mean trait value and its variance remain optimal—an environment‐driven parallel to the above phenotype‐driven “degraded arrows” scenario. Specifically, the height of the fitness peak can become lower (arrow “E” pointing downward on the legend to Figure 3) as a result of, for example, decreasing habitat quality or quantity, or resource availability (Pleasants & Oberhauser, 2013). In this sort of change, the optimal phenotypes can be thought of as having a lower maximal lifetime fitness or, in the case of density dependence, a lower maximal density or population size. Clear examples include declining bird populations in response to habitat modification (Fuller et al., 1995) and the effects of phosphorus/nitrogen limitation in aquatic ecosystems (Elser et al., 2007).

### 4.7 Arrow‐induced target shift

Each of the above two triplets of scenarios focused on externally driven phenotypic changes (1–3) or externally driven environmental changes (4–6). The final triplet of scenarios invokes internally driven eco‐evolutionary or eco‐plasticity feedbacks between phenotypes and environments, each being dependent on the other. Most obviously, when two species coevolve antagonistically, each is part of the “environment” for the other, such that evolution in one of the species shifts the fitness landscape for the other. For instance, the evolution of parasite resistance in a host can favor better evasion of host immunity by the parasite (Dybdahl & Lively, 1998). This coevolution results in a new trait optimum for the host's immune traits, some distance from their current state. Fitness for each party in the interaction thus remains suboptimal while each species' trait mean chases a continually moving optimum determined by the other species' moving trait mean. Similarly, the “environment” for a species includes its own density, potentially leading to cycles of trait values and densities, as has been argued for side‐blotched lizards specifically (Sinervo, Svensson, & Comendant, 2000) and intrasexual competition in general (Kokko & Rankin, 2006).

### 4.8 Arrow‐induced target contraction

Extending from the just‐described interaction‐driven changes in trait means and optima, changes in population size can affect the strength of stabilizing selection. For example, classic offspring size/number trade‐offs can be weakened by the availability of additional food resources (Crossner, 1977; McAdam, Boutin, Dantzer, & Lane, 2019). Changes in per capita resource availability resulting from changes in population size could then alter stabilizing selection on clutch size. More specifically, if per capita resources were more limited for extreme phenotypes (fewer resources for their particular phenotypes), competition could further decrease their fitness and thereby increase stabilizing selection around the local optima (Hendry, 2004). Such eco‐evolutionary feedbacks involving trait variances and nonlinear selection are not as well known as those involving trait means and directional selection, but are logically possible.

### 4.9 Arrow‐induced target degradation

When population density is too high (too many arrows), or individuals are too voracious (overly impactful arrows), the population might experience fitness declines owing to depleted resources, attraction or spread of local predators or pathogens, or excessive secreted waste or allelopathic products. As a result, the entire fitness landscape might sink overall (density dependence, arrow “E” in the legend to Figure 3) or, more specifically, wherever the arrows are most numerous or largest (frequency/density dependence).

In the case of “too many” arrows, very high densities of individuals can degrade the environment in ways that reduce mean absolute fitness, resulting in population declines (Ricker, 1954; de Roos & Persson, 2013). Such density‐dependent absolute maladaptation could be transient, or generate population cycles, if the resulting population decline leads to improved environmental conditions and population recovery. Examples include host–pathogen systems (Hochachka & Dhondt, 2000) and species characterized by “boom–bust” cycles (Uthicke, Schaffelke, & Byrne, 2009). In the case of “overly impactful arrows,” natural selection sometimes favors the evolution of increased per capita resource consumption, which can degrade the local environment without increased abundance. This increased per capita intake of resources can be favored by individual‐level selection even though it dramatically decreases population size (Anten & Vermeulen, 2016). This process, whereby natural selection leads to decreasing population sizes (Abrams, 2019), can potentially lead to population extinction—variously referred to as “Darwinian extinction” or “evolutionary suicide” (Gyllenberg, Parvinen, & Dieckmann, 2002; Rankin & López‐Sepulcre, 2005; Webb, 2003).

A fitness peak might also sink (arrow “E” pointing downward on the legend to Figure 3) when population size is small rather than large, even if individuals within that population are optimally adapted to the fitness landscape. Such a depression in fitness can occur when individual fitness is positively density dependent (i.e., Allee effect), wherein individual fitness is dependent on interactions with other individuals (Courchamp, Clutton‐Brock, & Grenfell, 1999).

## 5 ARROWS AND TARGETS IN THIS SPECIAL ISSUE

Here, we briefly describe the 19 papers in this Special Issue. The relevance of each paper to maladaptation and its corresponding archery scenario is summarized in Table 1. Tillotson, Barnett, Bhuthimethee, Koehler, and Quinn (2019) show that artificial selection created through fish hatchery practices can lead to maladaptation in natural settings (*biased arrows*), where wild and hatchery individuals coexist. Due to climate change (*moving target*), optimal spawning time is likely shifting later in the year (linked to hydrological and thermal conditions), contrasting hatchery practices (*biased arrows*) that favor earlier‐spawning fish. Fraser et al. (2019) found that maladaptation in wild brook trout occurred after just one generation of captivity. Maladaptation was both sex‐biased and more intense for populations with lower heterozygosity, providing important insights for conservation practices. Negrín Dastis, Milne, Guichard, and Derry (2019) used transplant experiments and theory to show that asymmetric selection together with immigration can contribute—via *biased arrows*—to the persistence of maladaptation within a copepod metapopulation.

**Table 1:** Arrows and targets in this issue

Author (Research approach)	Approach and maladaptation insight	Archery scenario and rationale
Bridle et al. (Modeling)	Adaptation was constrained at range limits when environmental gradients were steep between populations	*Imprecise arrows; damaged arrows.* Immigration across gradient produces excessive trait variance, decreasing mean population fitness and favoring drift rather than selection
Brady et al. (Empirical)	“Woodland” populations of frogs had low components of fitness and performance compared to “roadside” populations	*Moving target.* Woodland populations might be maladapted to infrequent but strong changes in environment (e.g., periodic disease outbreak) that are less common/protected against in roadside habitats
De León et al. (Empirical)	Human presence and food sources in Galapagos eroded niche diversity that has driven adaptive radiation, potentially undermining species future coexistence	*Target expansion* (not described in Figure 2) potentially leading to *arrow‐induced target contraction*. Newly available food resources (i.e., an expanding target) abundant to multiple species might reduce niche differentiation, making coexistence less likely for one or more species as the dominant species' effect is to contract the target for other species
Derry et al. (Synthesis; conceptual framework)	Conservation targets differ along a gradient from “adaptive state” to “adaptive process.” Such targets can yield maladaptation by accident or design	*Moving target* and *imprecise arrows.* Environmental change can move optimum beyond range of current population, especially for threatened populations. Adaptive state approaches can result in *moving target* problems, while adaptive process approaches can result in *imprecise arrows*
Fitzpatrick & Reid (Empirical)	For guppies, gene flow from mainstem to headwater streams can be a source of maladaptation but can also benefit adaptation to changing conditions	*Moving target* and *imprecise arrows.* Gene flow increases genetic variation (*imprecise arrows*) but can help populations evolve to stressors familiar to source populations; genetic variation does not necessarily aid adaptation to stressors unfamiliar to source and recipient populations
Fraser et al. (Empirical)	Captivity of wild brook trout can induce maladaptation after one generation and can differ between sexes	*Moving target* and possibly *retreating target.* Captive conditions differ from wild conditions (*moving target*) and require more time for adaptive responses and/or support lower maximum fitness (*retreating target*)
Geladi et al. (Empirical)	Fish populations declined following river impoundment and predator introductions. Despite these stressors, populations showed no clear evidence of adaptive responses	*Moving target and/or biased arrows*. An abrupt change in environment might have shifted optima far from previous position, constraining opportunity for adaptive responses, particularly if the “arrows” were precisely distributed
Gering et al. (Review)	Maladaptation is common in artificially selected organisms; domestication and feralization also mediate fitness in wild populations via gene flow and invasion dynamics	All nine scenarios are evaluated in light of domestication and feralization literature
Loria et al. (Review)	Negative demographic effects of pollution intensify across generations	*Moving/retreating/degrading targets* and *damaged arrows.* Pollution shifts optima, and potentially shrinks and reduces their quality size and quality. Pollution can also *damage arrows* (e.g., DNA damage)
Lasky (Modeling)	Genetic load can be transient and later beneficial; competition mediates this outcome	*Imprecise arrows* can become precise following an instance of *moving target*
Martinossi‐Allibert et al. (Empirical)	The interplay between sexual and fecundity selection mediated (mal)adaptation to a stressful environment. Individual male tolerance to stress increased under sexual selection at the cost of population decline	*Moving target.* Sexual and fecundity selection were manipulated, forcing adaptation to multiple moving targets
Negrín Dastis et al. (Empirical; modeling)	Asymmetric selection and dispersal maintained maladaptation in a metapopulation of copepods distributed across habitats that vary in pH	*Biased arrows*. Asymmetric selection can bias local populations toward a trait optimum displaced from the optima of nearby habitats connected by dispersal
Poirier et al. (Empirical)	Demographic bottleneck in bighorn sheep caused inbreeding depression that was later reversed through translocation efforts resulting in genetic and demographic recovery	*Damaged arrows*. Inbreeding depression caused 40% reduction in female lamb overwinter survival
Robertson & Horváth (Empirical)	Artificial light attracts insects to oviposit in poor habitat (“evolutionary trap”). Broad‐spectrum light was the main driver of the trap, but light color can mediate the strength of attraction	*Moving target*. Evolutionary traps such as these represent a special type of *moving target*, where the environment changes in a duplicitous way
Singer & Paremsan (Empirical)	Butterflies colonized a novel host in patches cleared by logging and prescribed burns. Butterfly fitness increased—despite maladaptation to the novel host—because clearing/fire disturbance dramatically improved host suitability by extending its life span. But local butterflies remained maladapted to their novel host relative to imported butterflies adapted to the same host in nearby, undisturbed habitats	*Moving target.* Logging and fire added a novel target: use of a novel host that immediately provided higher fitness than the traditional host, the use of which remained as a target in unlogged patches interdigitated with clearings. *Biased arrows.* Gene flow out of logged patches increased maladaptive acceptance of the novel host in unlogged patches, where it reduced butterfly fitness
Svensson & Connallon (Modeling)	Frequency‐dependent selection made adapting to environmental change more difficult in most cases	*Moving target* and *arrow‐induced target shift*. Environmental change was the moving target. Frequency‐dependent selection shifted the target as the frequency of a phenotype affected its fitness
Tillotson et al. (Empirical and modeling)	Hatchery practices selected for earlier reproduction, countering presumed direction of selection from climate change	*Biased arrows and moving target*. Hatchery selection biased the population toward an artificially created optimum. Climate change is expected to create a moving target that runs counter to the direction optimized by hatchery practices
Tseng et al. (Empirical)	Resource evolved faster than consumer to warming conditions, rendering consumer maladapted	*Arrow‐induced target shift*. Evolution of algae to warming shifted its *Daphnia* consumer's target because evolved algal traits made exploitation by *Daphnia* less effective
Walters & Berger (Empirical)	Dispersal and spatial scale mediated maladaptation/time to extinction when environments changed	*Moving target*. Maladaptation was induced through environmental change

Note

Contributed articles to this Special Issue are summarized in terms of their relation to the nine scenarios of maladaptation described in this paper. Assignments are not mutually exclusive, and some studies could be described in terms of other archery scenarios.

Walters and Berger (2019) developed theory related to a climate change (*moving target*) and local adaptation, focusing on how the ubiquity of local adaptation influences a population's ability to track a *moving target* across a preexisting environmental gradient. They showed that spatial scale and dispersal both mediated maladaptation when populations were locally adapted across the gradient. Bridle, Kawata, and Butlin (2019) modeled maladaptive trait variance caused by gene flow. They showed that maladaptation is more intense when an environmental gradient between connected populations is steep. Interestingly, this *imprecise arrows* scenario produced by dispersal and gene flow can arise through plasticity (e.g., if phenotypes are induced by natal environmental before dispersal). Thus, *imprecise arrow*s can occur even if genotypes are optimal for the receiving environment, reflecting excessive trait variance even in the absence of excessive genetic variance.

Derry et al. (2019) provide a conceptual framework for maladaptation insights (especially related to *moving targets)* in conservation, noting how different management strategies can be viewed in terms of a gradient of desired outcomes, from adaptive states (low trait variation close to target) to adaptive processes (high trait variation to respond to *moving targets*). They also conducted a meta‐analysis to compare success (e.g., long‐term fitness) across a variety of evolutionary‐minded conservation strategies such as genetic rescue and hybridization. Poirier, Coltman, Pelletier, Jorgenson, and Festa‐Bianchet (2019) show the effectiveness of one of these strategies—genetic rescue via translocation—applied to a bighorn sheep population that had previously undergone a demographic bottleneck (*damaged arrows*) but recovered following translocation efforts.

Gering, Incorvaia, Henriksen, Wright, and Getty (2019) reviewed literature on domestication and feralization in light of *all nine archery scenarios*. They found that maladaptation is common, noting that population histories and local environmental variation mediate fitness and that domestication and feralization can impact wild population fitness and cause maladaptation. Loria, Cristescu, and Gonzalez (2019) also conducted a survey of the literature, this time on adaptation to environmental pollutants. They found that most studies in this context concerned various *moving targets* or *damaged arrows,* but assayed the phenotypes of individuals, making it difficult to scale up to population fitness. Demographic studies often found negative population growth persisting or even intensifying over time, suggesting persistent absolute maladaptation despite adaptive shifts in phenotypes.

Lasky (2019) developed theory around the dynamics of gene flow‐induced maladaptation (caused by increased genetic variation) and environmental change in a community context. Rates of gene flow and differences in evolutionary pace between species mattered for maladaptation and also facilitated ecosystem stability (mediated by competitor release). Thus, initial maladaptation from *imprecise arrows* can lead to adaptation following a *moving target* scenario, and competition can influence these outcomes. Fitzpatrick and Reid (2019) demonstrated the context dependency of gene flow using an empirical assessment. For guppies, gene flow from mainstems to headwater streams increased genomic variation and improved tolerance to experimentally‐induced stress (*moving target*), but only for a stress that was familiar to the source (mainstem) populations (thus, also *imprecise arrows*).

Svensson and Connallon (2019) developed much‐needed theory on applied aspects of frequency‐dependent selection, which can decrease or increase population mean fitness. They asked when and to what extent frequency‐dependent selection affects population persistence, particularly in the context of environmental change (*moving target*). In most cases, frequency‐dependent selection compromised a population's ability to evolve in response to environmental change.

Tseng, Bernhardt, and Chila (2019) used a transplant experiment in a two‐species community context to evaluate the effects of warming on a consumer–resource system. They found evidence for an *arrow‐induced target shift*: Evolution by the faster‐evolving resource (algae) resulted in maladaptation for its consumer (*Daphnia*). Traits that were adaptive for algae in warmer conditions negatively affected *Daphnia*, which were slower to evolve. Martinossi‐Allibert et al. (2019) evolved seed beetles with different mating regimes to dissect the effects of sexual selection, fecundity selection, and male–female coevolution on individual and population mean fitness. Sexual selection on males increased female fitness across all environments (i.e., “good genes”). Sexual selection on males, with fecundity selection removed, increased male fitness in stressful environments (but at a cost to female fecundity). These results illustrate how demography in novel environments (*moving target*) can reflect the sex‐specific and sometimes conflicting influences of sexual and fecundity selection.

De León et al. (2019) found higher diversity of Darwin's finches in urban areas. Finch diet in those areas was biased toward human food items. The authors suggest that this new, prevalent food resource associated with humans increased niche breadth (what might be called a *target expansion*), potentially merging the diversity of narrower niches (*arrow‐induced target contraction*) that generate and maintain diversification, thereby raising concern for the persistence of this classic example of adaptive radiation. Geladi et al. (2019) used a 100‐year time series from collections to study morphology of fishes affected by the construction of the Panama Canal, which changed a river into an impounded lake and spurred novel predator introductions (*moving target*). These changes caused local fishes to decline, yet produced little morphological change. Thus, despite apparently strong selective forces and absolute fitness decline, morphological traits did not seem to evolve.

Singer and Parmesan (2019) described a long‐term study of butterflies that colonized a novel host in logged and burned forest patches. There, the butterflies remained adapted to their traditional host and maladapted to the novel host in six separate host‐adaptive traits, including alighting bias, geotaxis, clutch size, and offspring performance. Despite these maladaptations caused by a *moving target*, insect fitness increased on the poorly defended, novel host compared to the traditional host that was still used in adjacent unlogged patches. This increase in fitness occurred because of dramatic changes in host suitability mediated by logging and fire. Populations using the novel host boomed for 20 years. Biased dispersal out of the logged patches (“matching habitat choice”) reduced fitness of the dispersing individuals and drove maladaptive evolution of host preference in undisturbed patches (*biased arrows*) where butterflies still used their traditional host.

Brady et al. (2019b) found that previously described patterns of maladaptation in roadside frogs appeared to be reversed in a more northerly metapopulation. There, roadside populations outperformed woodland populations, suggesting that maladaptation patterns are complex across space and time and that human‐modified environments (*moving target*) can at times support positive outcomes for local species. In another empirical study of *moving target* maladaptation, Robertson and Horváth (2019) took a closer look at an evolutionary trap caused by artificial light, which attracts insects to oviposit in poor habitat. While light color had some influence on attractiveness (blue and red lights were least attractive), the main source of this evolutionary trap was broad‐spectrum, unpolarized light.

## 6 CONCLUSION

Although we have a penchant for studying adaptation, maladaptation is common in the natural world. We suggest that increased focus on maladaptation will improve understanding in applied evolution—where studies are often concerned with reduced fitness, whether as an outcome to be avoided (e.g., when conserving threatened populations) or achieved (e.g., when managing pests or combatting disease). For instance, applied questions guided by maladaptation perspectives might help us better understand the limits of natural selection and the influence of the forces counteracting it. As maladaptation‐focused approaches grow, so too should our capacity for general insights into the prevalence and degree of the various causes of maladaptation. For instance, what environmental and population characteristics are likely to conduce maladaptation versus adaptation, and to what extent? Are some scenarios of maladaptation more common than others? And if so, are they more severe? The distribution of studies within this Special Issue nominally suggests that moving target scenarios are among the most prevalent (or at least the most studied), followed next by imprecise and then biased arrows. What does this distribution say about the other scenarios —are they understudied or merely uncommon?

Much remains to be discovered about the distribution and dynamics of maladaptation, particularly in applied contexts, where a focus on maladaptation is perhaps most essential. We hope this collection of studies, together with the conceptual framework presented here, will spur future work to develop this understanding of applied evolution, balancing our knowledge of maladaptation with that of adaptation.

## ACKNOWLEDGEMENTS

This paper and the idea for this Special Feature are the product of a working group on maladaptation funded by the Canadian Institute of Ecology and Evolution and by the Quebec Centre for Biodiversity Science. We thank the Gault Nature Reserve for providing an ideal setting for productive working group meetings.

## CONFLICT OF INTEREST

None declared.
